# Surfaces of gymnastic equipment as reservoirs of microbial pathogens with potential for transmission of bacterial infection and antimicrobial resistance

**DOI:** 10.3389/fmicb.2023.1182594

**Published:** 2023-04-20

**Authors:** Mengge Zhang, Yanan Ma, Hai Xu, Mingyu Wang, Ling Li

**Affiliations:** State Key Laboratory of Microbial Technology, Microbial Technology Institute, Shandong University, Qingdao, Shandong, China

**Keywords:** gymnastic equipment, microbiome, bacterial infection, microbial community structure, antimicrobial resistance

## Abstract

Gymnastic equipment surfaces are shared by many people, and could mediate the transfer of bacterial pathogens. To better understand this detrimental potential, investigations on the reservoirs of bacterial pathogens and antimicrobial resistance on the surfaces of gymnastic equipment were performed by analyzing the bacterial community structures, prevalence of viable bacteria, and presence of antimicrobial resistance on both indoor and outdoor gymnastic facilities. The results of high-throughput 16S rDNA amplicon sequencing showed that Gram-positive bacteria on the surfaces of indoor gymnastic equipment significantly enriched, including the opportunistic pathogen *Staphylococcus* strains, while Enterobacteriaceae significantly enriched on surfaces of outdoor gymnastic equipment. The analysis of α-diversities showed a higher richness and diversity for bacterial communities on the surfaces of gymnastic equipment than the environment. Analysis of β-diversities showed that the bacterial communities on the surfaces of gymnastic equipment differ significantly from environmental bacterial communities, while the bacterial communities on indoor and outdoor equipment are also significantly different. Thirty-four bacterial isolates were obtained from the surfaces of gymnastic equipment, including three multidrug *Staphylococcus* and one multidrug resistant *Pantoea*. In particular, *Staphylococcus hemolyticus* 5–6, isolated from the dumbbell surface, is a multidrug resistant, hemolytic, high- risk pathogen. The results of quantitative PCR targeting antibiotic resistance related genes (*intI1*, *sul1* and *bla*_TEM_) showed that the abundances of *sul1* and *bla*_TEM_ genes on the surfaces of gymnastic equipment are higher than the environment, while the abundances of *sul1* gene on indoor equipment are higher than outdoor equipment. These results lead to the conclusion that the surfaces of gymnastic equipment are potential dissemination pathways for highly dangerous pathogens as well as antimicrobial resistance, and the risks of indoor equipment are higher than outdoor equipment.

## Introduction

Transmission of infectious diseases can be accelerated by contamination of pathogens on the surfaces people access. Bacterial transfer rate during routine household activities has been assessed, showing that despite having low transfer efficiency, there are still a sizable number of transmitted bacteria from surfaces to human skin (up to 10^6^ cells; Rusin et al., 2002). To provide convenience for people’s exercises, a significant amount of public gymnastic equipment has been established in communities, gymnasiums, and universities worldwide. This could potentially increase the risks of bacterial contamination and dissemination. During exercises, one of the most common pathways of bacterial infection transmission is direct skin-to-equipment contact. People sweat as a result of their bodies warming up, which creates a favorable environment for microbial proliferation on the skin. In addition, physical stress damages the body’s exterior defenses, opening the door for pathogen invasion (Grosset-Janin et al., 2012).

Antibiotics are the primary treatments for bacterial infections (Laxminarayan et al., 2016). Nevertheless, along with the widespread use of antibiotics in medical and agricultural sectors from the mid-20th century to the early 21st century, antimicrobial resistance (AMR) has emerged and become currently one of the most urgent medical threats (Browne et al., 2021). Antibiotic resistance genes (ARGs) can be disseminated between bacteria *via* horizontal gene transfer mechanisms and spread from environmental bacteria to pathogens, accelerating dissemination of AMR. This dissemination has been evaluated in a number of environmental settings. For instance, hospitals and wastewater treatment plants are ARG reservoirs and hotspot for ARG dissemination, in which the mobile resistome and dissemination dynamics have been extensively studied in recent years (Peter et al., 2020; Zhu et al., 2023). Even in clean environments such as university campus air, multidrug resistant bacterial pathogens in particulate matters were reported (Hu et al., 2023). With these observations, suspicions are raised that public gymnastic equipment that are relatively clean but shared by many people may also become pathways for the transmission of AMR and pathogens. However, despite the high frequency of gymnastic equipment use in university campus, the assessment of the spread of infectious diseases *via* shared gymnastic facilities is rarely reported, and the spread of bacterial infection by these facilities has not been given enough attention.

In order to explore whether gymnastic equipment increases risks for AMR transmission, in this work, we applied microbiomics, bioinformatics, microbiology, and molecular biology methods to explore the potential for transmission of bacteria and resistance *via* gymnastic equipment, providing a comparative analysis of the structures of microbial community, the distribution of pathogenic bacteria, as well as the abundance of ARGs on different gymnastic equipment surfaces. Overall, this investigation emphasized the role and risk of gymnastic equipment as a pathway for the transmission of bacterial infections, which had previously been underappreciated.

## Materials and methods

### Sample collection and preparation

Samples were collected from the outdoor sports field and the indoor gymnasium at Qingdao University of Science and Technology. The outdoor equipment includes parallel bars, horizontal bars, climbing bars and monkey bars. The indoor equipment includes dumbbell, barbell, crunch bench and leg extension. Samples collected from the outdoor ground and indoor door were used as the controls for the outdoor and indoor samples, respectively. Sterile cotton swabs soaked in sterile saline were used to repeatedly wipe the gymnastic equipment surfaces that frequently come into contact with the human body. The cotton was then transferred into sterile centrifuge tubes. Three and four replicates were set up for outdoor and indoor control samples, respectively. All samples were stored at 4°C until processing. One milliliter of sterile were added to the centrifuge tubes, which were vortexed for 30 s to prepare the suspension for processing.

### 16S rDNA amplicon sequencing and bioinformatics analysis

Total DNA was extracted from swab samples by Plant Genome DNA Extraction Kit (Tiangen Biotech Co., Ltd., Beijing.,China). 16S rDNA amplicon sequencing was performed on the Illumina HiSeq2500 sequencer (Illumina, Inc., San Diego, CA, US). Sequencing libraries were generated using TruSeq® DNA PCR-Free Sample Preparation Kit (Illumina, Inc., San Diego, CA, United States). Using FLASH v1.2.7 and QIIME v1.7.0, the high-quality clean tags from the raw sequence data were obtained (Caporaso et al., 2010). Chimeras were removed using UCHIME algorithm (Edgar et al., 2011). Using Uparse v7.0.1001 (with default sequence similarity of 97%), Operational Taxonomic Units (OTU) classification was determined (Edgar, 2013). The raw sequencing datasets are available at NCBI SRA repository (accession PRJNA942587).

### Bioinformatical analysis and statistics

The analysis of microbial community composition was performed *via* the RDP classifier 2.2 algorithm in Silva database (Version 132) (Wang et al., 2007; Quast et al., 2013). Alpha diversity and beta diversity were calculated by QIIME v1.7.0 and displayed with R software (Version 2.15.3). NMDS, ANOSIM and MRPP analyses were performed with the Vegan package on the R platform. LEfSe (LDA effect size) analysis was performed using LEfSe. The comparison of bacterial community structure and genes abundance of bacteria were analyzed *via* two-tailed *t*-test. Tukey and Wilcoxon tests were used to analyze alpha-diversities.

### Bacteria isolation and antimicrobial susceptibility test

Sample suspension was inoculated on blood agar plates and MacConkey plates respectively, followed by incubation at 37°C for 16 h. Genomic DNA of individual colonies was extracted using the Plant Genomic DNA Extraction Kit (Tiangen Biotech Co., Ltd., Beijing, China). Subsequently, the universal primer pair 27F (5′ - AGAGTTTGATCCTGGCTCAG - 3′) /1492R (5′ - GGTTACCTTGTTACGACTT - 3′) was used to amplify 16S ribosomal RNA gene by PCR for further sequencing and the taxonomic identification of these isolates. Antimicrobial susceptibility test of the isolates was determined according to the Clinical & Laboratory Standards Institute (CLSI) guidelines using the Kirby-Bauer disk diffusion method (Clinical and Laboratory Standards Institute, 2018).

### Quantitative real-time PCR

Genes associated with antimicrobial resistance, including mobile genetic element *intI1* and the three most common ARGs (*intI1*, *sul1*, *bla*_TEM_) were determined with qRT-PCR. Primers used in this study are the same as previously reported and shown in Supplementary Table S1 (Barraud et al., 2010; Li et al., 2021; Wei et al., 2023). Two-step Real Time RT-PCR was performed with a StepOnePlus real-time PCR system (Applied Biosystems, Waltham, MA, United States), according to previously published literature for PCR program (Haenelt et al., 2023). Standard curves for the target genes were constructed using pMD19-T vector. Each sample was analyzed with three biological replicates.

## Results

### Microbial community structures on gymnastic equipment surfaces

**Figure 1:**
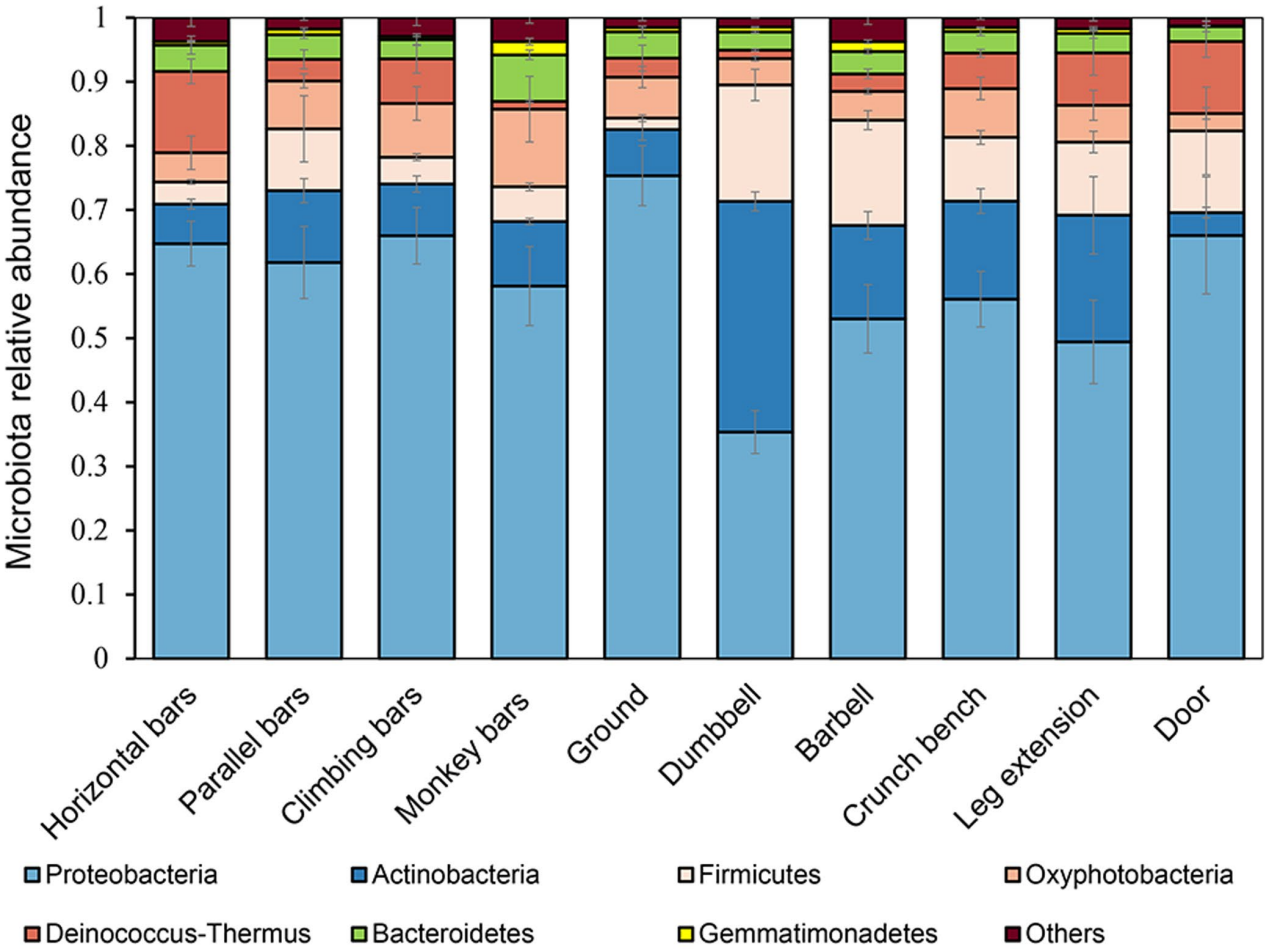
Microbial community structures on the surfaces of gymnastic equipment.

Bacterial microbiomes were determined for each gymnastic equipment sample by high throughput 16S rDNA amplicon sequencing (Figure 1). At phylum level, the main bacterial groups were Proteobacteria, Actinobacteria, Firmicutes, Oxyphotobacteria, Deinococcus-Thermus, Bacteroidetes, Gemmatimondadetes, accounting for more than 96% in the microbial community (Figure 1). Among them, Proteobacteria, the most abundant phylum in the environment also had the highest proportion in our research (Yan et al., 2017). Overall, there were obvious structural differences between the microbial communities on the surfaces of indoor equipment (such as barbells, crunch bench machines, dumbbells, and leg extensions) and those on outdoor equipment (monkey bars, parallel bars, horizontal bars, climbing bars). Compared to outdoor equipment, on the surfaces of indoor equipment, the percentage of Gram-positive bacteria from the phyla Actinobacteria (*p* = 7.04 × 10^−4^) and Firmicutes (*p* = 1.78 × 10^−4^) increased dramatically, but the percentage of Gram-negative bacteria from the phylum Proteobacteria (*p* = 2.00 × 10^−3^) decresed significantly. This may be explained by the proposal that the impact of human is smaller on outdoor equipment because of wind and rain.

At the genus level, observed abundant bacteria on gymnastic equipment include environmentally safe bacteria such as *Methylbacillum*, *Sphingobacterium*, *Hydrophilus*, and *Cynobacterium*, along with opportunistic pathogens like *Acinetobacter*, *Staphylococcus* and *Enterobacter*. Interestingly, *Methylbacillum* and *Sphingobacterium* were prevalent in microbiomes from samples collected from the ground at a high rate of 21.84 ± 3.85% (*n* = 4) and 12.64 ± 6.49% (*n* = 4) respectively, significantly higher than other samples (*n* = 28, *p* = 1.89 × 10^−22^; *n* = 28, *p* = 5.89 × 10^−8^). Meanwhile, the levels of human opportunistic pathogenic *Staphylococcus* found on indoor equipment surfaces were 4.67 ± 3.53% (n = 12), much greater than the 0.47 ± 0.48% in samples collected from the ground (n = 4, *p* = 0.036).

### Analysis of microbial community α-diversities on the surfaces of gymnastic equipment

**Figure 2:**
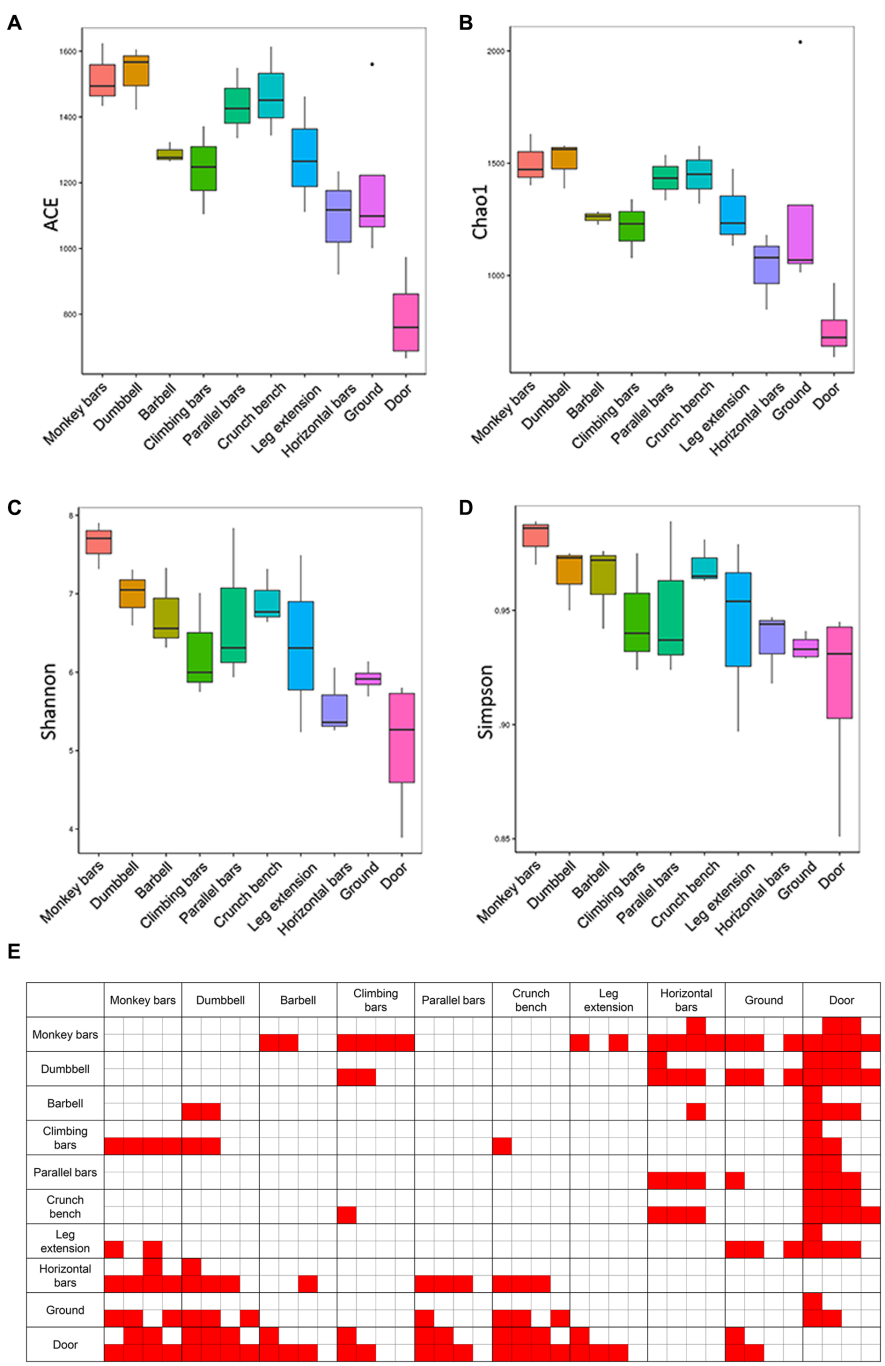
Abundances and diversities of microbial communities on the surfaces of gymnastic equipment. **(A)** ACE estimators. **(B)** Chao1 estimators. **(C)** Shannon indices. **(D)** Simpson indices. **(E)** Statistical tests of α-diversity indices. Red, represents *p*-values <0.05. In each comparison box from left to right are comparisons of the ACE estimators, Chao1 estimators, Shannon indices and Simpson indices. The top four small boxes in each comparison box mark the results of the Tukey test and the bottom four small boxes mark the results of the Wilcoxon test.

Alpha diversity analysis, including Chao1, ACE, Shannon and Simpson indices, was performed to calculate microbial community richness and diversities (Figure 2). The Shannon and Simpson indices were employed to measure community diversity, and the Chao1 and ACE indices were chosen to measure community richness. As shown in Figure 2, the microbiome in monkey bars had the highest richness and diversity of any piece of equipment, while horizontal bars had the lowest. Besides, the microbial community composition on the surfaces of gymnastic equipment was generally more diverse and rich than those on the surface of the ground and door, assessed with Tukey and Wilcoxon tests, suggesting human activities indeed altered the microbial community.

### Similarities between microbial communities

**Figure 3:**
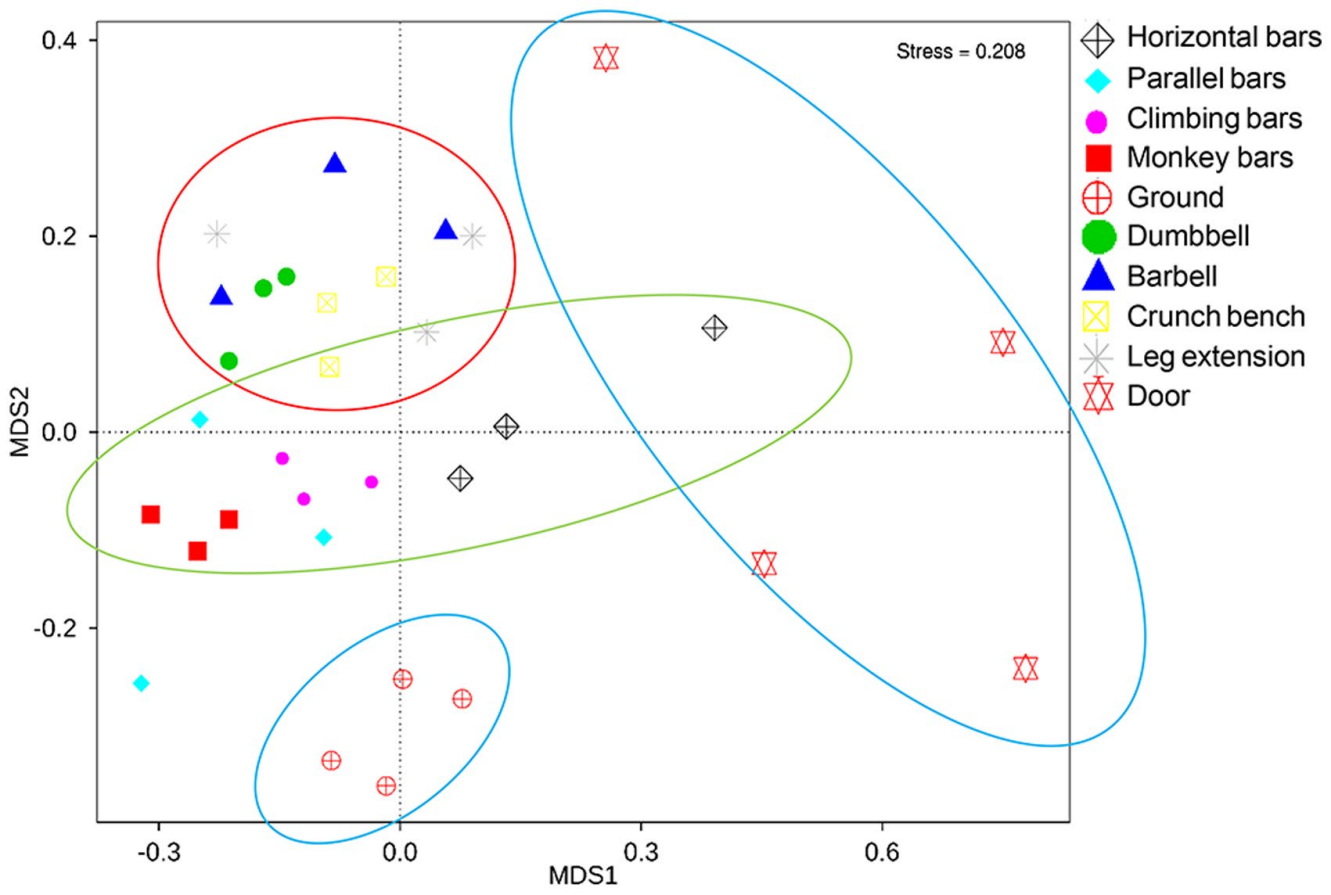
NMDS analysis of microbial community on the surfaces of gymnastic equipment. Red circle, represents indoor equipment; green circle, represents outdoor equipment; blue circle, represents controls.

Non metric multidimensional calibration method (NMDS) is a common technique to determine similarities and grouping of high-dimensional data in ecology or microbial ecology (Shepard, 1980). With each point representing a sample, we were able to use NMDS to assess the similarities between microbiomes collected from gymnastic facilities. It can be shown in Figure 3 that a significant difference is present between the surface microbiota of outdoor and indoor equipment, as well as between the surface microbiota of gymnastic equipment and the environmental microbiota (ANOSIM, *p* = 0.001; MRPP *p* = 0.001). This is also a clear support that human activities while using gymnastic equipment has a significant impact on microbiome structures.

### Enrichment of microbes on the surfaces of different equipment

**Figure 4:**
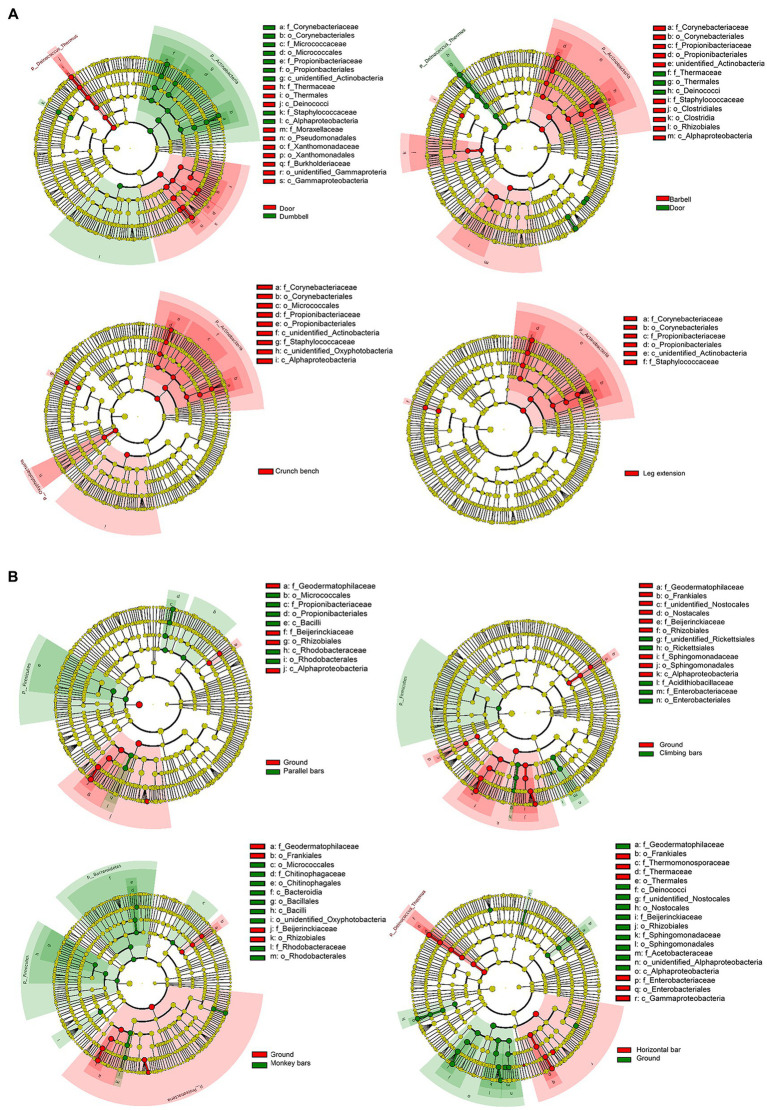
Bacterial enrichment on the surfaces of different gymnastic equipment. **(A)** Comparison of door and indoor equipment. **(B)** Comparison of ground and outdoor equipment.

In order to identify the microbes enriched on the surface of gymnastic equipment, LEfSe, a technique to analyze enriched bacteria in various microbiomes, was applied (Figure 4). Door and ground sample were used as controls for indoor gymnastic equipment and outdoor gymnastic equipment, respectively. Compared with the control microbiota, human-origin opportunistic pathogens, such as *Staphylococcus*, *Rickettsia*, and *Enterobacter*, showed a significant increase, but ambient bacteria decrease to varying degrees on the surface of gymnastic equipment (Figure 4A). In particular, *Staphylococcus* increased significantly on the surface of indoor equipment, while *Enterobacter* bacteria increased significantly on the surface of outdoor equipment (Figure 4B). This is a strong signal that gymnastic equipment may transmit human pathogens.

### Identification of opportunistic pathogens and determination of antimicrobial resistance

With the investigations on microbiomes, we found that on both indoor and outdoor equipment, there is an enrichment of opportunistic pathogens from humans. To further demonstrate that the enriched bacteria were indeed viable and potentially pathogenic, microorganisms from the samples were isolated, and antimicrobial resistance was analyzed. A total of 34 isolates were isolated, including 24 strains of *Bacillus* spp., 5 strains of *Staphylococcus*, 2 strains of *Pantoea*, 1 strain of *Sporosarcina*, 1 strain of *Enterobacter*, and 1 strain of *Pseudomonas* (Table 1). Of the five *Staphylococcus* strains isolated, four was haemolytic, with a clear haemolytic ring (beta-haemolysis) on blood agar plate, demonstrating their strong pathogenicity.

**Table 1:** Strains isolated from gymnastic equipment surfaces.

Location	Gymnastic equipment	Strains	16S rDNA identification
Indoor	Horizontal bars	1–1	*Bacillus* sp.
		1–2	*Bacillus mycoides*
		1–3	*Bacillus* sp.
		1–4	*Pantoea agglomerans*
	Parallel bars	2–1	*Bacillus altitudinis*
		2–2	*Staphylococcus* sp.
	Climbing bars	3–1	*Bacillus megaterium*
		3–2	*Bacillus* sp.
	Monkey bars	4–1	*Bacillus altitudinis*
		4–2	*Bacillus* sp.
		4–3	*Bacillus* sp.
Outdoor	Dumbbell	5–1	*Bacillus subtilis*
		5–2	*Bacillus* sp.
		5–3	*Bacillus* sp.
		5–4	*Bacillus* sp.
		5–5	*Bacillus oleronius*
		5–6	*Staphylococcus haemolyticus*
		5–7	*Sporosarcina* sp.
		5–8	*Bacillus megaterium*
		5–9	*Bacillus* sp.
		5–10	*Bacillus altitudinis*
		5–11	*Bacillus* sp.
		5–12	*Bacillus subtilis*
		5–13	*Bacillus* sp.
	Barbell	6–1	*Staphylococcus haemolyticus*
		6–2	*Staphylococcus hominis*
		6–3	*Pantoea* sp.
		6–4	*Bacillus* sp.
	Crunch bench	7–1	*Brevibacillus* sp.
		7–2	*Enterobacter hormaechei*
		7–3	*Bacillus* sp.
		7–4	*Bacillus* sp.
	Leg extension	8–1	*Staphylococcus hominis*
Control	Ground	9–1	*Pseudomonas oryzihabitans*

Antimicrobial susceptibility was tested for all isolated strains of *Staphylococcus* and *Pantoea* (Tables 2, 3). For *Staphylococcus* strains, antimicrobial resistance was tested against chloramphenicol (CHL), erythromycin (ERY), kanamycin (KAN), trimethoprim (TMP), co-trimoxazole (SXT), ciprofloxacin (CIP), rifamycin (RIF), and tetracycline (TET; Table 2). For *Pantoea* strains, apart from CHL, TMP, CIP, and TET, seven extra antibiotics for Gram-negative strains including streptomycin (STR), nalidixic acid (NAL), ampicillin (AMP), cefepime (FEP), ceftazidime (CAZ), imipenem (IPM), tigecycline (TGC) were tested (Table 3). Four of the five strains of *Staphylococcus* were isolated from indoor equipment and most were multidrug resistance. It’s worth nothing that *Staphylococcus haemolyticus* 5–6, in addition to being hemolytic, were also resistant to CHL, ERY, KAN and CIP. For the two *Pantoea* strains*, Pantoea agglomerans* 1–4, isolated from the horizontal bars was sensitive to all antibiotics, while *Pantoea* sp. 6–3, isolated from the barbell, is multidrug resistant bacteria. This finding suggested that the pathogenic bacteria isolated from gymnastic equipment, especially the bacteria carried on indoor equipment with pathogenicity and multidrug resistance, threaten human health.

**Table 2:** Antimicrobial susceptibility test and hemolytic test of *Staphylococcus* spp.

		Parallel bars	Dumbbell	Barbell		Leg extension
	*Staphylococcus aureus* ATCC25923	*Staphylococcus* sp. 2–2	*Staphylococcus haemolyticus* 5–6	*Staphylococcus haemolyticus* 6–1	*Staphylococcus hominis* 6–2	*Staphylococcus hominis* 8–1
CHL	S	S	R	S	S	S
ERY	I	R	R	R	I	S
KAN	S	S	R	S	R	S
TMP	S	S	S	S	S	S
SXT	S	S	S	S	S	R
CIP	S	S	R	I	S	S
RIP	S	S	S	S	S	S
TET	S	S	S	R	S	S
Hemolytic	No	Yes	Yes	Yes	Yes	No

CHL, Chloramphenicol; ERY, Erythromycin; KAN, Kanamycin, TMP, Trimethoprim; SXT, Sulfamethoxazole; CIP, Ciprofloxacin; RIP, Rifampicin; TET, Tetracycline. R, resistant; S, susceptible; I, intermediate.

**Table 3:** Antimicrobial susceptibility test of *Pantoea* spp.

Antibiotics		Horizontal bars	Barbell
	*Escherichia coli* ATCC25922	*Pantoea. agglomerans* 1–4	*Pantoea* sp. 6–3
CHL	S	S	R
STR	S	S	S
NAL	S	S	I
TMP	S	S	S
AMP	S	S	I
CIP	S	S	S
FEP	S	S	S
CAZ	S	S	I
IPM	S	S	S
TGC	S	S	S
TET	S	S	S

CHL, Chloramphenicol; STR, Streptomycin; NAL, Nalidixic acid; TMP, Trimethoprim; AMP, Ampicillin; CIP, Ciprofloxacin; FEP: Cefepime; CAZ, Ceftazidime; IPM: Imipenem; TGC: Tigecycline, TET, Tetracycline. R, resistant; S, susceptible; I, intermediate.

### Abundance of ARGs on the surfaces of gymnastic equipment

To further characterize the potential for transmission of AMR in gymnastic equipment, the abundances of the following genes were quantitatively analyzed: 16S rDNA gene (to represent total bacteria levels), the integron integrase gene *intI1* (to characterize mobile genetic elements capable of transmitting ARGs; Hall, 2012), the sulfonamide resistance gene *sul1* (Razavi et al., 2017), and the β-lactam antimicrobial resistance gene *bla*_TEM_ (Bonomo, 2017).

**Figure 5:**
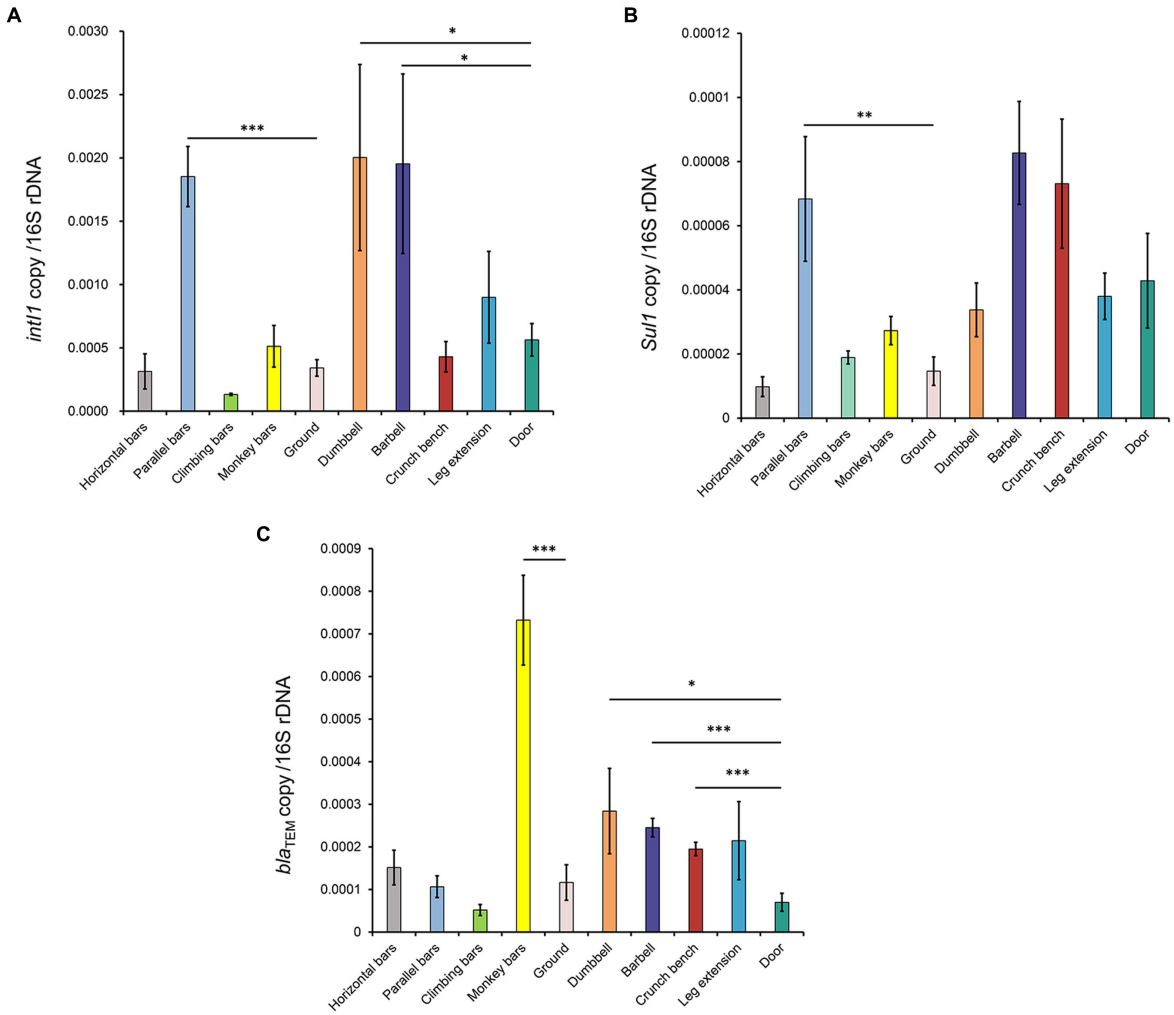
Normalized abundance of ARGs on the surfaces of gymnastic equipment. **(A)** *intI1*. **(B)** *sul1*. **(C)** *bla*_TEM_. *, *p* < 0.05; **, *p* < 0.01; ***, *p* < 0.001.

As shown in Figure 5, the normalized ARG levels increased on different gymnastic equipment surfaces. The integron integrase gene *intI1* significantly increased on the surfaces of parallel bars, dumbbell, and barbell. The abundances of *sul1* significantly increased on the surfaces of the parallel bars, while the abundances of *bla*_TEM_ increased significantly on the surfaces of monkey bars, dumbbell, barbell, and crunch bench, indicating that the abundance of ARGs on the surfaces of these sports equipment was higher than that in the environment. When comparing the ARGs abundance of indoor and outdoor equipment, it was shown that the abundance of *sul1* on the surfaces of gymnastic equipment was higher than that of outdoor equipment. Taken together, these data suggest that sports equipment can significantly enrich for ARGs, facilitating their transmission and potentially causing the spread of resistance.

## Discussion

In universities, gymnastic facilities are accessible to the entirety of students and faculty, which often include 1,000’s of people. Considering the frequency of use and direct human contact with equipment, the surfaces of these public gymnastic equipment may act as transmission pathways of pathogenic bacteria, leading to the spread of infection. In this study, the analysis of microbial communities on the surface of gymnastic equipment suggested that the use of gymnastic equipment can indeed significantly change the microbial community structures on the surfaces of equipment, resulting in enrichment of specific pathogenic bacteria on the surface of sports equipment, such as *Staphylococcus* and *Enterobacter*. The rapid increase of bacterial resistance has become one of the urgent problems in the field of medicine and health in the 21st century (Li et al., 2018). In this study, all the 4 strains of hemolytic *Staphylococcus* isolated from indoor equipment were drug-resistant bacteria, among which 3 strains were multidrug-resistant bacteria. In particular, *Staphylococcus hemolyticus* 5–6 isolated from the dumbbell surface is a multidrug-resistant, hemolytic *Staphylococcus* strain, being resistant to 4 of the 8 antibiotics. We summarized the percentage of antibiotic resistance for each isolate and the link between the percentage and presence-absence of resistance genes (see Supplementary materials Table S2, S3; Supplementary Figures S1–S4). By further quantitatively analyzing the abundance of drug resistance genes, we found that not only potential pathogens but also common ARGs (*bla*_TEM_, *sul1* and *intI1*) were enriched on the surfaces of gymnastic equipment. This further illustrates the risk of gymnastic equipment in the transmission of bacterial infections.

This study has demonstrated that gymnastic equipment increases risks in the process of bacterial infection transmission. However, it does not mean that we should limit or even avoid using communal gymnastic equipment. On the one hand, the human skin is a powerful barrier against external bacteria. On the other hand, the overall benefits of exercise to improve physical health and immunity outweigh the risk of bacterial infections. However, for certain groups of people, such as those with traumatic skin injuries, those with compromised immune systems and those who are temporarily sick, using public gymnastic equipment for exercise may expose them to the threat of bacterial infections, leading to increased health and economic burdens. To address the above risk of bacterial infection dissemination *via* gymnastic equipment, we recommend that risk assessment and caution is needed when using public sports equipment for high-risk groups, especially those with surface wounds or lower immunity. Secondly, gymnastic equipment, especially indoor equipment, should be disinfected on a regular basis to prevent the dissemination of bacterial infection and antimicrobial resistance.

## Data availability statement

The raw sequencing datasets are available at the Sequence Read Archive (SRA) under BioProject number PRJNA942587.

## Author contributions

MZ and YM performed experiments. MZ wrote the manuscript. HX, MW, and LL critically revised the manuscript. LL conceived of the study and oversaw the project. All authors contributed to the article and approved the submitted version.

## Funding

This work was supported by the National Natural Science Foundation of China [grant number 8227060729].

## Conflict of interest

The authors declare that the research was conducted in the absence of any commercial or financial relationships that could be construed as a potential conflict of interest.

## Publisher’s note

All claims expressed in this article are solely those of the authors and do not necessarily represent those of their affiliated organizations, or those of the publisher, the editors and the reviewers. Any product that may be evaluated in this article, or claim that may be made by its manufacturer, is not guaranteed or endorsed by the publisher.
