# Supplementary material for: Surfaces of gymnastic equipment as reservoirs of microbial pathogens with potential for transmission of bacterial infection and antimicrobial resistance
Source: Front Microbiol. 2023 Apr 20;14:1182594. doi: 10.3389/fmicb.2023.1182594 (PMC10157288; doi:10.3389/fmicb.2023.1182594)
Supplement: Supplementary file 1 [file Data_Sheet_1.docx]

Supplementary Material

# Supplementary Tables

**Table S1**. **Primers used in qPCR reactions.**

| Target gene | Primer sequence (5´-3´) | Product size·(bp) |
| --- | --- | --- |
| *intI1* | F: CGCACCGGAAACATCGCTGCAC | 196 |
|  | R: TGAAGTTCCGCCGCAAGGCTCG |  |
| *bla*_TEM_ | F: CTTTATCCGCCTCCATCCAGTCTA | 247 |
|  | R: TGCCTGCCCATCATTAGCC |  |
| *sul1* | F: CGCACCGGAAACATCGCTGCAC | 162 |
|  | R: TGAAGTTCCGCCGCAAGGCTCG |  |
| 16S rDNA | F: CCCAGATGGGATTAGCTTGT | 106 |
|  | R: TCTGGACCGTGTCTCAGTTC |  |

**Table S2**. **Link between antimicrobial resistance and antibiotic resistance genes.** For isolates with antimicrobial resistance, the corresponding antibiotic resistance genes were examined by PCR. -: antibiotic resistance gene was absent; /: detection of resistance gene was not performed. Since isolates 5-6, 6-2 and 8-1 lost viability, detection of their resistance genes was not performed.

| Antibiotics | Antibiotic resistance genes | | |
| --- | --- | --- | --- |
|  | 2-2 | 6-1 | 6-3 |
| ERY | *ermB* | *ermB* | - |
| AMP | / | / | *bla*_NDM_ |

**Table S3**. **The results of antibiotic resistance percentage for five *Staphylococcus* sp. and two *Pantoea* sp..**

|  | Antibiotics and percentage | | | | | | | | | | |
| --- | --- | --- | --- | --- | --- | --- | --- | --- | --- | --- | --- |
| *Staphylococcus* | CHL | ERY | KAN | TMP | SXT | CIP | RIF | TET |  |  |  |
|  | 1/5 | 4/5 | 2/5 | 0/5 | 1/5 | 2/5 | 0/5 | 1/5 |  |  |  |
| *Pantoea* | CHL | STR | NAL | TMP | AMP | CIP | FEP | CAZ | IPM | TGC | TET |
|  | 1/2 | 0/2 | 1/2 | 0/2 | 1/2 | 0/2 | 0/2 | 1/2 | 0/2 | 0/2 | 0/2 |

# Supplementary Figures


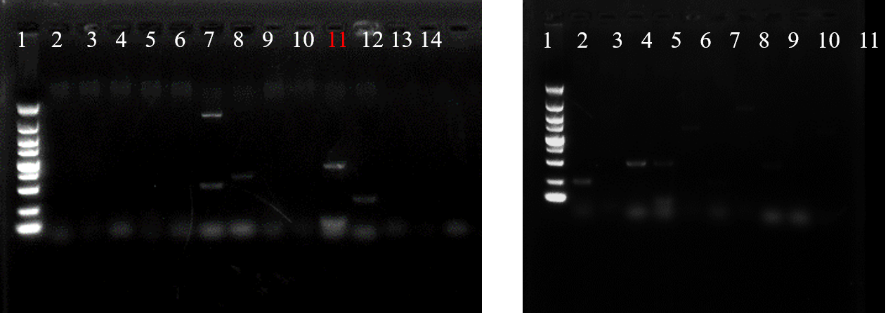


**Figure S1.** **The agarose gel electrophoresis for *Staphylococcus haemolyticus* 6-1**. Left pannel, lane 1-14: marker, *qnrA*, *qnrB*, *qrnC*, *qnrD*, *qnrS*, *oqxA*, *oqxB*, *qepA*, *intI1*, *ermB*, *mphE*. Right pannel, lane 1-5: marker, *tetA*, *tetB*, *tetC*, *tetD*, *tetM*, lane 6-11: negative control. Red, gene confirmed by sequencing.

**
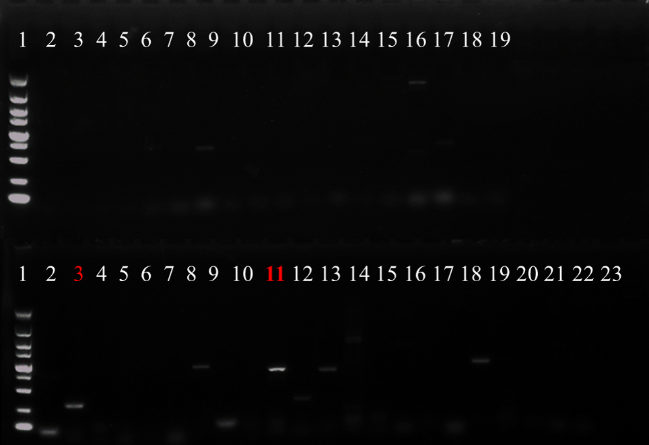
**

**Figure S2.** **The agarose gel electrophoresis for *Pantoea* sp.6-3 and *Staphylococcus* sp.2-2**. Upper, lane 1-10 (*Pantoea* sp.6-3): marker, *qnrA*, *qnrB*, *qrnC*, *qnrD*, *qnrS*, *oqxA*, *oqxB*, *qepA*, *intI1*, lane 11-19: negative control. Lower, lane 1-9 (*Pantoea* sp.6-3): marker, *bla*_KPC_, *bla*_NDM_, *bla*_VIM_, *bla*_OXA_, *bla*_IMP_, *bla*_CTX_, *bla*_TEM_, *bla*_SHV_, lane 10 -12 (*Staphylococcus* sp. 2-2): *intI1*, *ermB*, *mphE*, lane 13-23: negative control. Red, gene confirmed by sequencing.


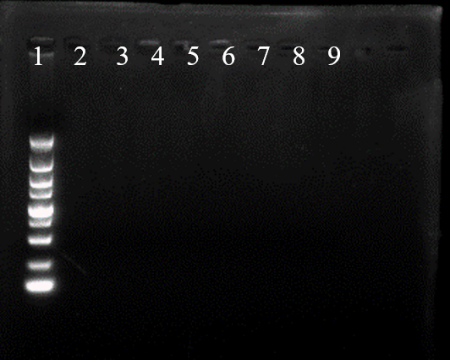


**Figure S3.** **The agarose gel electrophoresis for *Pantoea* sp.6-3.** Lane 1-5: marker, *catI*, *floR*, *cmlA*, *catB*, lane 6-9: negative control.


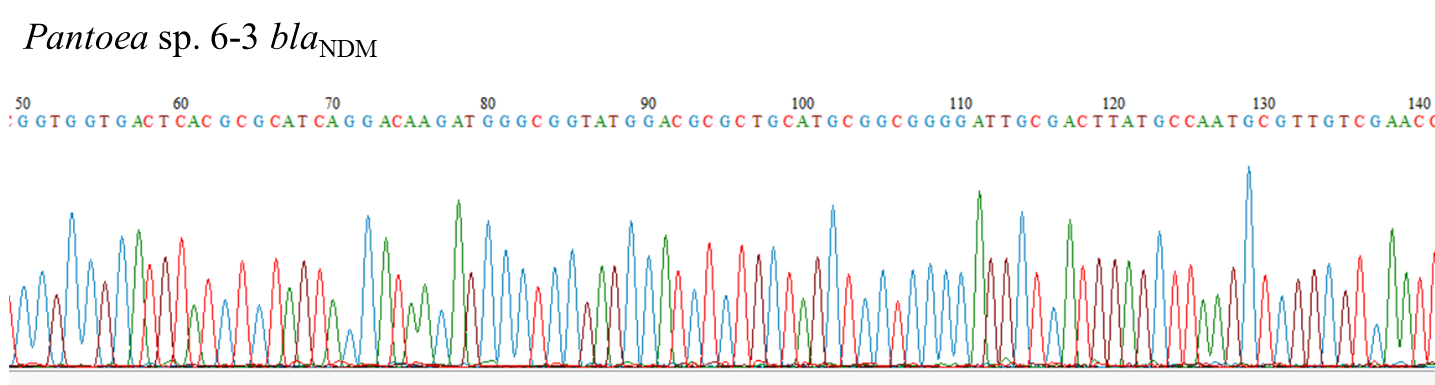

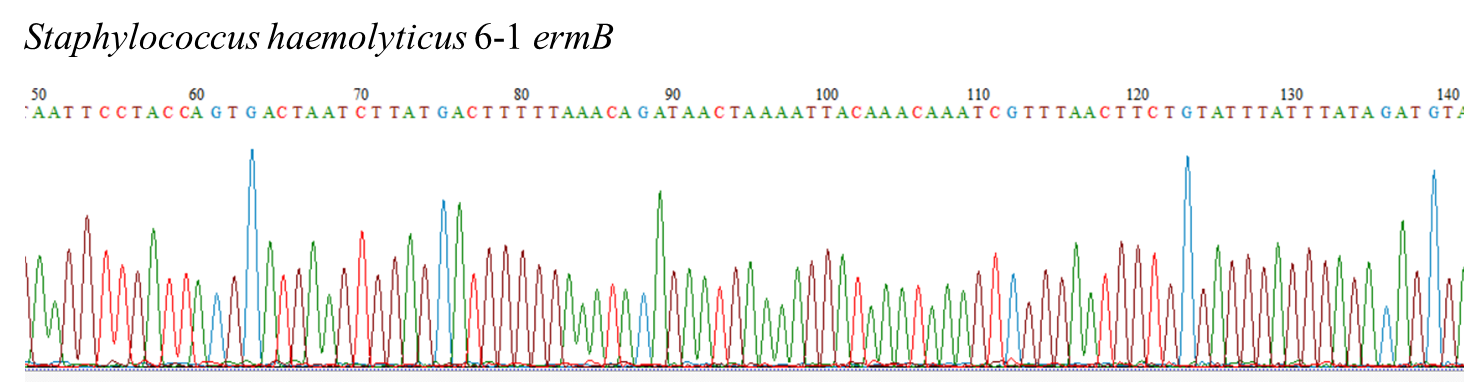

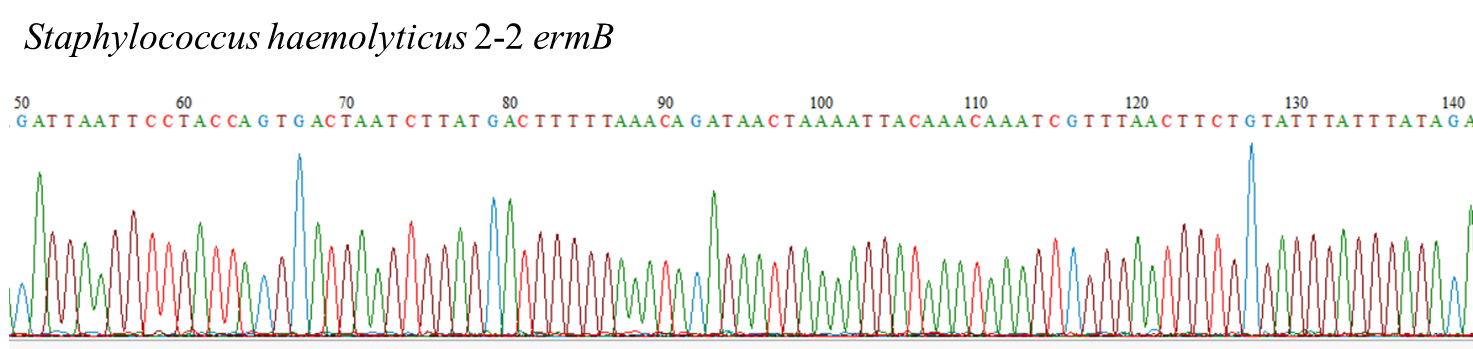


**Figure S4.** **The results of sequencing of detected antibiotic resistance genes.**
